# Supplementary material for: Therapeutic Drug Monitoring of Anti-TNFα Inhibitors: A Matter of Cut-Off Ranges
Source: Pharmaceutics. 2023 Jun 27;15(7):1834. doi: 10.3390/pharmaceutics15071834 (PMC10386140; doi:10.3390/pharmaceutics15071834)
Supplement: Supplementary file 1 [file pharmaceutics-15-01834-s001.zip › pharmaceutics-2454838-supplementary.pdf]

Supplementary.

Data analysis

Data were analysed using descriptive statistics. Continuous variables, normally distributed, were reported as means and standard deviations and compared with the t test. Continuous variables not following normal distribution were analysed by the Mann-Whitney U test and presented as medians and interquartile range (IQR). The normality of IFX and ADM plasma concentrations distribution for each method was checked using the Kolmogorov-Smirnov test. Using the Passing-Bablok regression, the quantitative results from the two experiments were compared. To quantify the agreement between IFX and ADM levels from the two assays, the Pearson's correlation coefficient was calculated whereby a value of 1 represents complete agreement. Linear regression has been used to assess the slope between assays, and R^2 was calculated to check the goodness of the fit. A R^2 value of 1 indicates a perfect linear correlation while a R^2 value of 0 translates no correlation. Bland-Altman analysis was used to measure assays agreement and bias between each assay. Briefly, the difference between the two measurements was presented on the Y-axis and the average of the two measurements on the X-axis. Ideally, a flat line result confirms agreement between two assays (20). In this graphical method, differences between each pair of assays are plotted against averages of the pair and bias is then calculated. 95% of the agreed limits were subsequently calculated. For qualitative comparison, weighted kappa statistics were determined after stratification of results by therapeutic interval (<3 mg/L, ≥3 to 7 mg/L and ≤7 mg/L for IFX concentrations and <5 mg/L, ≥5 to 10 mg/L and ≤10 mg/L for adalimumab concentrations, according to the manufacturer's instruction). The agreement between the results obtained with the two assays was assessed using Cohen's Kappa coefficient which takes the value of (i) zero if there is no more agreement between the two tests than can be expected by chance, (ii) 1 if there is perfect agreement. Kappa results are commonly interpreted as follows: values lower than 0.2 as indicating slight agreement, values between 0.2 and 0.4 as fair, values between 0.4 and 0.6 as moderate, values between 0.6 and 0.8 as substantial and values greater than 0.8 as almost

perfect agreement while negative Kappa indicates poor agreement. The level of statistical significance was set at 0.05.
